# Enrichment of Membrane Proteins for Downstream Analysis Using Styrene Maleic Acid Lipid Particles (SMALPs) Extraction

**DOI:** 10.21769/BioProtoc.4728

**Published:** 2023-08-05

**Authors:** Benedict Dirnberger, Dagmara Korona, Rebeka Popovic, Michael J. Deery, Helen Barber, Steven Russell, Kathryn S. Lilley

**Affiliations:** 1 Cambridge Centre for Proteomics, Department of Biochemistry, University of Cambridge, Cambridge, United Kingdom; 2 Department of Genetics, University of Cambridge, Downing Street, Cambridge, United Kingdom; 3 MRC Toxicology Unit, Gleeson Building, University of Cambridge, Cambridge, United Kingdom

**Keywords:** Styrene Maleic Acid Lipid Particles (SMALPs), *Drosophila melanogaster*, Affinity Purification, Nicotinic Acetylcholine Receptor (nAChR), Mass Spectrometry, Ligand-Receptor Interaction, Native Membrane Protein Extraction

## Abstract

Integral membrane proteins are an important class of cellular proteins. These take part in key cellular processes such as signaling transducing receptors to transporters, many operating within the plasma membrane. More than half of the FDA-approved protein-targeting drugs operate via interaction with proteins that contain at least one membrane-spanning region, yet the characterization and study of their native interactions with therapeutic agents remains a significant challenge. This challenge is due in part to such proteins often being present in small quantities within a cell. Effective solubilization of membrane proteins is also problematic, with the detergents typically employed in solubilizing membranes leading to a loss of functional activity and key interacting partners. In recent years, alternative methods to extract membrane proteins within their native lipid environment have been investigated, with the aim of producing functional nanodiscs, maintaining protein–protein and protein–lipid interactions. A promising approach involves extracting membrane proteins in the form of styrene maleic acid lipid particles (SMALPs) that allow the retention of their native conformation. This extraction method offers many advantages for further protein analysis and allows the study of the protein interactions with other molecules, such as drugs. Here, we describe a protocol for efficient SMALP extraction of functionally active membrane protein complexes within nanodiscs. We showcase the method on the isolation of a low copy number plasma membrane receptor complex, the nicotinic acetylcholine receptor (nAChR), from adult *Drosophila melanogaster* heads. We demonstrate that these nanodiscs can be used to study native receptor–ligand interactions. This protocol can be applied across many biological scenarios to extract the native conformations of low copy number integral membrane proteins.

## Background

Membrane proteins represent more than 60% of all drug targets; yet their insoluble behaviour makes it very difficult to study native interactions with drug molecules (Yin and Flynn, 2016; Liu et al., 2020). The development of methods to effectively extract membrane proteins and ensure their continued functionality is thus of paramount importance. The lipid bilayer surrounding membrane proteins is essential for structural integrity, stability, and ligand binding (daCosta et al., 2013). Moreover, within this lipid environment, most membrane proteins exist as part of multi-protein complexes with other membrane-embedded proteins and peripherally associated proteins. In order to understand the correct binding interactions of membrane proteins with other molecules, it is thus necessary to retain their lipid environment in a manner that does not disrupt their interacting protein partners. The requirement to keep protein and lipid associated in a native and functionally relevant state is extremely challenging, as most membrane protein extraction methods rely on the use of ionic and zwitterionic detergents for complete solubilization. Furthermore, the application of typical non-ionic and non-denaturing detergents such as Triton and Tween result in significant disruption of lipid–protein interactions that affect the native conformation of proteins (Stetsenko and Guskov, 2017).

The development of methods for extracting membrane proteins from lipid bilayers using detergents and introducing them into artificial lipid nanodiscs has facilitated a much better characterization of receptor–ligand interactions (Denisov and Sligar, 2016). The use of detergents generally employed to solubilize membrane proteins, however, leads to destabilization, aggregation, and misfolding, and their use is therefore not compatible with this type of analysis (Loo et al., 1996).

The use of small organic compounds such as styrene maleic acid lipid particles (SMALPs) allows detergent-free extraction of membrane proteins in their local lipid environment, providing a promising technique for investigating receptor–ligand interactions under native conditions (Lee et al., 2016). As a copolymer, styrene maleic acid (SMA) has a high affinity for membranes and is readily incorporated into lipid bilayers. SMA polymerizes to form a *girdle*, which traps disc-shaped pieces of membrane containing the membrane proteins, their interacting partners, and the lipid environment in a native state (Xue et al., 2018). The individual discs vary in size and shape, with an average size of 10 nm. It has been shown that membrane proteins with 37 transmembrane helices can be recovered in SMALP discs (Lee et al., 2016). The Piezo, a mechanosensory ion channel protein that contains 37 predicted transmembrane helices in *Drosophila melanogaster*, for example, has been shown to be readily incorporated into SMALP discs (Korona et al., 2022). Maintenance of the lipid environment in these nanodiscs is particularly important, since loss of lipids surrounding membrane proteins can lead to changes in measured binding affinities (Martens et al., 2018; Gault et al., 2020). To date, SMALP nanodisc technology has been applied to many different biological systems (Lee et al., 2016; Teo et al., 2019; Korona et al., 2022).

Recently, we have established a protocol that employs SMALP extractions to create nanodiscs, containing still functionally active low abundance membrane receptor protein complexes. The protocol is compatible with subsequent affinity enrichment, enabling identification of their constituent parts by mass spectrometry while retaining activity, such that native receptor–ligand interactions can also be interrogated (Korona et al., 2022). Thus, the combination of detergent-free SMALPs extraction coupled with mass spectrometry analysis provides a potential route for characterizing native membrane receptor complexes (Sobotzki et al., 2018; Kalxdorf et al., 2021).

We applied this protocol to study components of nicotinic acetylcholine receptors (nAChRs). These neurotransmitter receptors belong to a large class of insecticide targets located in synaptic plasma membranes (Ihara et al., 2020). These pentameric cys-loop ligand-gated ion channels consist of either only α-subunits or α- and β-subunits, with ligand-binding sites located between two α-subunits or between α- and β-subunits. The structural model of *D. melanogaster* nAChR contains 10 highly conserved subunits that assemble in various combinations to form the active receptors (Lu et al., 2022). Using our approach, we were able to elucidate which subunits assemble to form functional receptor complexes (Korona et al, 2022). We also demonstrated the ability of the five nAChRs within these discs to interact with α-Bungarotoxin (α-BTX), a small peptide toxin found in snake venom, which is known to bind and modulate the activity of this receptor.

The following protocol provides a method to study the native interaction of *D. melanogaster* adult head nAChRs extracted into SMALPs. This method can be readily transferred to other biological systems for the effective native extraction of membrane protein complexes and to investigate the interactions of these proteins with other molecules under native conditions. Our protocol will therefore have a major impact on future drug interaction studies that will identify potential binding to other native membrane proteins.

## Materials and reagents

1. *w^1118^ (D. melanogaster*) [FlyBase, FBal0018186, Bloomington drosophila stock center (BDSC): 3605]
2. *nAChRα6^FSVS^ (D. melanogaster*) (Korona et al., 2022)
3. Styrene maleic acid copolymer (SMA) 3:1 [supplied by Prof. Tim Dafforn, University of Birmingham, UK, personal communication https://www.birmingham.ac.uk/staff/profiles/biosciences/dafforn-tim.aspx, and commercially available, for example, SMALP 300 (Cube Biotech, catalog number: 18200)]
4. Pierce^TM^ quantitative fluorometric peptide kit (Thermo Scientific, catalog number: 23290)
5. Trypsin/Lys-C Mix (Promega, catalog number: V5073)
6. C-18 material (Thermo Scientific, catalog number: 84,850)
7. Pierce C-18 spin tips (Thermo Fisher Scientific, catalog number: 84,850)
8. α-Bungarotoxin (α-BTX) (Abcam, catalog number: ab120542)
9. Antibody
  Anti-ATPase alpha 1 (Abcam, catalog number: ab2872)
  Anti-mouse ECL peroxidase labeled (Merck, catalog number: GENA931-1ML)
  Anti-GFP (goat monoclonal) (Abcam plc, catalog number: Ab252881)
  Anti-rat IgG (goat polyclonal) (Sigma-Aldrich, catalog number: A9037)
10. Isotonic lysis buffer (see Recipes)
11. SMALP solution (see Recipes)
12. Coupling buffer (see Recipes)
13. Tris-buffer or TBS (see Recipes)
14. TBS-T buffer (see Recipes)
15. Blocking solution (see Recipes)
16. Laemmli buffer (see Recipes)
17. Sample buffer (see Recipes)

**Chemical reagents**

1. Carbachol (Insight Biotechnology Ltd, catalog number: CAS 51-83-2)
2. Cyanogen bromide (CNBr)-activated Sepharose affinity beads (Sigma-Aldrich, catalog number: C9 142-5G)
3. cOmplete^TM^ protease inhibitor (Merck, catalog number: 11836170001)
4. Marvel dried skimmed milk (5% solution)
5. ECL chemiluminescent detection solution (GE Healthcare, catalog number: 45-000-999)
6. Tween 20 (Sigma-Aldrich, catalog number: P1379-25ml)
7. Ammonium bicarbonate (NH_4_HCO_3_) (Sigma-Aldrich, catalog number: 09830-500G)
8. Bromophenol blue (Sigma-Aldrich, catalog number: 114391-5G)
9. Coomassie brilliant blue G250 (Sigma-Aldrich, catalog number: 1154440025)
10. Ethanol (Sigma-Aldrich, catalog number: 1070172511)
11. Acetic acid (Sigma-Aldrich, catalog number: A6283-2.5l)
12. Sodium acetate (NaCH_3_CO_2_) (Sigma-Aldrich, catalog number: 32319-500G-R)
13. Sodium hydrogen carbonate (NaHCO_3_) (Sigma-Aldrich, catalog number: 1063290500)
14. Sodium chloride (NaCl) (Sigma-Aldrich, catalog number: S9888-25G)
15. Hydrogen chloride (HCl) (Sigma-Aldrich, catalog number: 320331-500ML)
16. HEPES (N-2-hydroxyethylpiperazine-N’-2-ethanesulfonic acid) (Sigma-Aldrich, catalog number: PHR1428-1)
17. Aluminum sulfate-(14-18)-hydrate (Sigma-Aldrich, catalog number: 368458-500G)
18. Methanol (Sigma-Aldrich, catalog number: 322415-2L)
19. Ortho-phosphoric acid (Sigma-Aldrich, catalog number: 345245-500ML)
20. EDTA (Ethylenediaminetetraacetic acid) (Sigma-Aldrich, catalog number: 03695-250G)
21. Tris(hydroxymethyl)aminomethane (Sigma-Aldrich, catalog number: 1070897600)
22. Sucrose (Sigma-Aldrich, catalog number: S0389-500G)
23. Glycine (Sigma-Aldrich, catalog number: G7126-100G)
24. Acetone (Sigma-Aldrich, catalog number: 179124-500ML)
25. Acetonitrile (ACN) (Sigma-Aldrich, catalog number: 34851-1L)
26. Dithiothreitol (DTT) (Sigma-Aldrich, catalog number: D9760-500MG)
27. Iodoacetamide (IAA) (Sigma-Aldrich, catalog number: I1149-25G)
28. Sodium dodecyl sulfate (SDS) (Sigma-Aldrich, catalog number: 8170341000)
29. Formic acid (FA) (Sigma-Aldrich, catalog number: 695076-100ML)
30. Methanol (Sigma-Aldrich, catalog number: 34860-2.5L-R)
31. HPLC water (Sigma-Aldrich, catalog number: 270733-2.5L)
32. Glycerol (Sigma-Aldrich, catalog number: G5516-100ML)

## Equipment

1. Sieve 200 mm diameter 400 micron (Endecotts, catalog numbers: 1201124)
2. Sieve 200 mm diameter 800 micron (Endecotts, catalog numbers: 1201125)
3. 2 mL Dounce homogenizer (DWK Life Sciences Limited, catalog number: 357422)
4. Nanodrop (DeNovix, catalog number: DS-11 FX+)
5. Beckman coulter optima^TM^ Max-XP Ultracentrifuge (Beckman Coulter, High Wycombe)
6. V-32 Vortex Mixer (GEM Scientific, catalog number: GERT-V-32)
7. Speed Vac (CentriVap Benchtop Centrifugal Vacuum Concentrator with acrylic lid, catalog number: 7810030)
8. Beckman coulter TLA 55 55 K RPM S/N 1601300 fixed angle rotor (Beckman Coulter, High Wycombe)
9. Microfuge tube polypropylene (Beckman Coulter, High Wycombe, catalog number: 357448)
10. Mini-Protean TGX precast gels (Bio-Rad Laboratories, catalog number: 456-1084)
11. Nitrocellulose membrane (Bio-Rad Laboratories, catalog number: 1704158)
12. Trans-Blot Turbo Transfer Pack (Bio-Rad Laboratories, Inc, catalog number: 1704158)
13. CL-XPosure films (Thermo Scientific, catalog number: 10465145)
14. X-ray developer (Protec GmbH, catalog number: 1170-1-8000)
15. Q Exactive Orbitrap mass spectrometer (Thermo Fisher Scientific, Waltham, MA, USA)
16. Dionex Ultimate 3000 RSLC nanoUPLC (Thermo Fisher Scientific, Waltham, MA, USA)
17. Reverse-phase nano Easy-spray column (Thermo Fisher Scientific, Waltham, MA, USA)

## Software

1. Proteome Discoverer 2.3 (Thermo Fisher Scientific, RRID:SCR_014477)

## Procedure

**Workflow overview**

The procedure starts with the separation of *D. melanogaster* heads for downstream enrichment of cellular membranes. Soluble proteins are separated from membrane proteins by means of centrifugation. Pelleted membrane proteins are solubilized in SMA 3:1 and incubated at room temperature to form the SMALPs. After the ultracentrifugation step, the supernatant contains a heterogeneous mixture of nanodiscs with a size between 5 and 15 nm that could be further examined using transmission electron microscopy. In order to target specific membrane proteins, an affinity purification is performed. In the example we give here, the α-BTX peptide is coupled to Sepharose beads and used to purify nAChRs. If necessary, the degree of enrichment of nAChRs is analyzed using western blot. In order to be able to identify the subunits of the receptor, the samples are digested using trypsin/lys-C mix and applied to LC–MS/MS. After analysis, a comparison against a non-enriched sample is performed to determine which protein subunits are specifically enriched.

**Membrane protein enrichment and incorporation in SMALPs**

The starting material can be adapted to the respective question. Material can range from human to bacterial cells, and from total tissues to whole organisms such as *D. melanogaster* (Gulati et al., 2014; Jamshad et al., 2015; Korona et al., 2022). The extraction protocol, however, must be adapted to the corresponding starting material. In this protocol, enriched heads from *D. melanogaster [w^1118^* and FSVS-tagged Dα6 (3xFLAG-StrepII-mVenus-StrepII)] are used to establish the protocol. *D. melanogaster* heads are obtained and separated according to Depner et al. (2014).

1. In a 50 mL Falcon tube, rapidly freeze approximately 6 g (approximately 6,000 flies; male 0.7 mg and female 1.0–1.3 mg, age dependent) of adult *D. melanogaster* in liquid nitrogen and vortex twice for 3 min. Cool the tube for 30 s in liquid nitrogen between vortexing.
2. Place two differently sized sieves into one another and transfer the flies into a liquid nitrogen–precooled 800-micron sieve. Separate heads from bodies by sieving. Collect heads in a second sieve with 400 microns in size. At this point, the heads can be stored at -80 °C for months before further extraction.
3. For cell lysis, add 1 mL of isotonic lysis buffer to approximately 0.8 g of separated heads. Mix the solution three times by vortexing and lyse the heads with 60 strokes in a 2 mL Dounce homogenizer with a pestle (Figure 1A).
4. Perform membrane protein preparation by differential centrifugation–based fractionation, as described in Depner et al. (2014). This allows for a better separation of membrane proteins from soluble proteins.
5. Perform a pre-cellular clearance by a centrifugation step at 200× *g* for 5 min. This step allows the removal of undisrupted cells, which are not broken during the extraction process.
6. Centrifuge the supernatants by a series of differential centrifugation steps: 1,000× *g* for 5 min, 3,000× *g* for 10 min, 5,000× *g* for 10 min, and 9,000× *g* for 15 min. Perform all steps in a 4 °C refrigerated centrifuge. After each spin, transfer the supernatant into a new centrifugation tube and use the membrane pellets for western blot analysis. By employing this differential centrifugation–based fractionation strategy, plasma membranes are partially separated from other endomembranes, increasing sensitivity and specificity of the system. With higher and higher spins, the fractions that do not contain the plasma membrane are obtained.
7. Western blot analysis reveals which fraction is enriched for the plasma membrane and thus should be used in downstream processing (Figure 1B).
8. Use fractions enriched in plasma membranes for the SMALP extraction. Resuspend membrane fractions (24–177 mg of wet pellet weight) in approximately 20–300 μL of 5% SMALP solution.
9. For efficient incorporation and formation of SMALPs, incubate fractions containing plasma membrane with 5% SMALP solution for 2 h at room temperature on a rocking platform. Finally, centrifuge at 100,000× *g* for 60 min at 4 °C and use the supernatant, which contains the SMALPs, for downstream analysis. SMALPs can be incubated on ice without precipitation, but low or rapid freezing can cause the nanodiscs to degrade.

**Immunoblotting**

Western blot analysis is used to investigate the enrichment of the plasma membrane proteins in fractions resulting from differential centrifugation. An anti-ATPase alpha 1 antibody is used to perform a western blot, acting as a plasma membrane marker (Figure 1B), or it can be used to determine the degree of enrichment of nAChRs.

1. After the centrifugation steps, load fractions on a 4%–15% SDS-PAGE and transfer onto a 0.2 μm nitrocellulose membrane.
2. Use 5% skimmed milk powder dissolved in TBS-T for blocking and incubate membranes for 16 h at 4 °C with the anti-ATPase alpha 1 antibody (1:1,000 concentrated in blocking solution) followed by anti-mouse ECL peroxidase labelled for 1 h. Either treat α-BTX-affinity enriched or unenriched protein samples with 1% DTT or leave untreated, boil at 60 °C for 8 min, separate by SDS-PAGE, and then transfer onto a nitrocellulose membrane.
3. Detect FSVS-tagged Dα6 (3xFLAG-StrepII-mVenus-StrepII) with anti-GFP (Figure 1C). This strain serves as a positive control for the successful enrichment of nAChRs using α-BTX affinity beads. Use 5% skimmed milk powder dissolved in TBS-T for blocking and incubate membranes for 16 h at 4 °C with the α-GFP antibody (1:1,000 concentrated in blocking solution) followed by anti-rat IgG antibody for 1 h.
4. Treat immunoblots with an ECL chemiluminescent detection solution exposed for 10 s to CL-XPosure films and visualize using an x-ray developer.
  **Figure 1.**
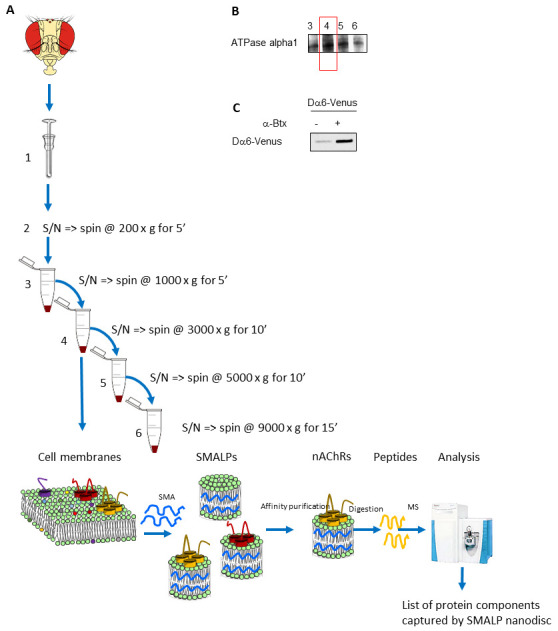
Overview of the protocol to enrich native plasma membrane proteins complexes and use styrene maleic acid lipid particles (SMALPs) enrichment and mass spectrometric analysis. A. In this example, *D. melanogaster* heads are used as a starting material. Cell membranes are first enriched using differential centrifugation. Membrane pellets are used to perform the SMALPs extraction. The plasma membrane–enriched fraction, in this case fraction 4, is mixed with SMA. The resulting nanodiscs containing the target protein of interest, in this case nAChRs, are further enriched using affinity beads coupled to α-BTX. Proteins within the discs are identified by firstly digesting to peptides using trypsin followed by LC–MS/MS. B. Western blot analysis to determine the fraction enriched in the plasma membrane using ATPase alpha 1 as marker. C. Western blot analysis to confirm the presence of the target membrane protein, in this example the fluorescent protein–tagged Dα6 nAChR subunit in enriched nanodiscs.

**Coupling procedure of α-BTX to affinity beads**

For this protocol, α-BTX-coupled beads are used to enrich for subunits of nAChRs. For other studies, alternative affinity enrichment strategies can be employed, such as specific antibodies used to target their corresponding membrane proteins. If ligands other than α-BTX are used, then ligand buffer solutions and coupling reactions must be optimized. Moreover, optimization of different linkers between affinity beads and ligands may be necessary if alternative ligands are employed. If antibodies or other ligands than α-BTX are used, the coupling and crosslinking reaction must be optimized. When using beads other than those described in this protocol, an optimization step should be performed.

1. Perform coupling of α-BTX to CNBr-activated Sepharose beads as described (Wang et al., 2003; Mulcahy et al., 2018).
2. Hydrate CNBr-activated Sepharose beads (0.25 g) in 1.25 mL of 1 mM HCl for 1 h at 4 °C on a rotator.
3. Centrifuge beads at 1,500× *g* for 5 min, remove the supernatant, and then wash the beads twice with 1 mL of coupling buffer.
4. Centrifuge beads at 1,500× *g* for 5 min and remove the supernatant. Resuspend α-BTX (1 mg) in 1 mL of coupling buffer and incubate together with the affinity beads at 4 °C for 16 h on a rotator.
5. Centrifuge beads at 1,500× *g* for 5 min. Remove the supernatant and keep it for measuring the coupling efficiency. At this time, the supernatant should contain no α-BTX. Determine coupling efficiency using a Pierce quantitative fluorometric peptide kit, according to the manufacturer’s instructions.
6. Block the beads with 1 mL of 0.2 M glycine in 80% coupling buffer at 4 °C for 16 h on a rotator.
7. Centrifuge the beads at 1,500× *g* for 5 min and wash with 1 mL of 0.1 M NaHCO_3_, 0.5 M NaCl, pH 8.0. Repeat this step with 1 mL of 0.1 M NaCH_3_CO_2_, 0.5 M NaCl, pH 4.0.
8. Wash the beads again in 1 mL of 0.1 M NaHCO_3_, 0.5 M NaCl, pH 8.0. After a final wash step with 1 mL coupling buffer, incubate the beads twice for 30 min in 1 mL of Tris-buffer (50 mM Tris, 150 mM NaCl, pH 8.0).
9. Centrifuge the beads at 1,500× *g* for 5 min and remove the supernatant.

**Enrichment of nAChRs by α-BTX pull-down**

If interacting molecules, such as toxin peptides other than α-BTX or antibodies, are used to enrich the desired membrane protein, the protocol should be adapted. The following protocol is developed with α-BTX, as this peptide has a high affinity for nAChRs (Dellisanti et al., 2007; Rahman et al., 2020).

1. Incubate SMALP discs (800–1000 μL of a 20–35 mg/mL protein extract, measured with a NanoDrop) with 200 μL of α-BTX-conjugated affinity beads for 16 h at 4 °C on a rotator.
2. Centrifuge the beads at 1,500× *g* for 5 min and wash two or three times, each for 10 min, with 1 mL of ice-cold TBS on a rotator at 4 °C.
3. Centrifuge the beads at 1,500× *g* for 5 min and selectively elute nAChRs twice with 100 μL of 1 M carbachol. Perform these steps on a rotator at RT.
4. Centrifuge the beads at 1,500× *g* for 5 min. The supernatant should be transferred into a new clean tube.
5. Add ice-cold 100% acetone to the samples at a volume of four times the sample volume and mix by vortexing. Leave proteins to precipitate for 16 h at -20 °C. An overnight precipitation using acetone allows the removal of contaminants including salts that may interfere with subsequent SDS-PAGE and analysis using western blotting. If further structural analysis of proteins is to be carried out using electron microscopy, this step may be skipped.
6. Centrifuge samples at 13,000× *g* for 15 min.
7. Remove the supernatant and dissolve precipitated proteins in Laemmli buffer. Heat the resulting sample at 60 °C for 8 min, load on Mini-Protean TGX precast gels, and resolve according to the manufacturer’s instructions. Perform protein staining according to Neuhoff et al. (1988). Fix gels in 40% (v/v) ethanol and 10% (v/v) acetic acid for 60 min, wash two times in water for 10 min, and stain for 16 h in Coomassie solution [0.1% (w/v) Coomassie brilliant blue G250, 5% (w/v) aluminum sulfate-(14-18)-hydrate, 10% (v/v) methanol, 2% (v/v) ortho-phosphoric acid]. Applying samples to an SDS-PAGE can be considered as an additional cleaning step.

**Sample preparation for liquid chromatography–mass spectrometry (LC-MS)**

Gel pieces are excised from the Coomassie stained gel lanes; proteolytic digestion, performed using a commercial available Trypsin/Lys-C mix, is performed as described (Shevchenko et al., 2006; Saveliev et al., 2013).

1. Immerse the gel pieces in 50 mM NH_4_HCO_3_/50% ACN and shake with a V-32 Vortex Mixer at maximum speed for 10 min. Remove the supernatant and repeat these steps with 100% ACN; finally, dry in a speed vac for 20 min.
2. Reduce samples with 10 mM DTT in 50 mM NH_4_HCO_3_ at 56 °C for 1 h. Remove DTT completely to avoid any inhibition effects on IAA.
3. Carry out alkylation with 50 mM IAA in 50 mM NH4HCO3 at room temperature without light for 45 min. Remove IAA.
4. Add 50 mM NH_4_HCO_3 _(fully cover the gel pieces); vortex for 5 min, centrifuge, and discard the solution.
5. Add 100% ACN to the gel pieces so they are completely covered with solution, shake for 10 min, and discard the solution.
6. Repeat these two steps (steps 4–5) and dry samples in a speed vac for 20 min. Add Trypsin/Lys-C buffer to the sample according to manufacturer’s instructions and incubate for 45 min on ice.
7. Next, add 30 μL of 25 mM NH_4_HCO_3_ and incubate samples at 37 °C for 16 h. Cover the gel pieces with 20 mM NH_4_HCO_3_ and shake with a V-32 Vortex Mixer at maximum speed for 10 min. Collect the supernatant containing peptides.
8. Next, cover the gel pieces with 50% ACN/5% FA and shake for 20 min. Again, collect the supernatant containing the peptides. Repeat this step of 50% ACN/5% FA addition and shaking for 20 min and collect the supernatant containing the peptides.
9. Combine all the supernatants together and dry in a speed vac until completely dry. Store samples at -20 °C.

**Peptide cleanup**

Peptides are desalted using C-18 stage tips according to Rappsilber et al. (2007).

1. Equilibrate C-18 material (three C-18 plugs are pasted in a 200 μL pipette tip) with 100 μL of methanol/0.1% FA.
2. Place the 200 μL pipette tip with an adaptor into a 2 mL centrifuge tube and centrifuge for 2 min at maximum speed. Remove the flowthrough.
3. Next, pipette 100 μL of 70% ACN and 0.1% FA to the 200 μL pipette tip and centrifuge for 2 min at maximum speed. Remove the flowthrough.
4. Finally, pipette 100 μL of 0.1% FA into the tip and centrifuge. Remove the flowthrough at this stage and repeat this step.
5. Place the 200 μL pipette tip into a new clean 2 mL tube. Resuspend the peptide pellets in 20 μL of fresh sample buffer to load the peptides onto the C-18 material.
6. Fully resuspend peptide samples by 15 min of vortexing. Then, carefully pipette peptides onto the C-18 material and, to ensure that the solution is in contact with the C-18 material, briefly centrifuge the 200 μL pipette tip [2–5 s at approximately 1,000 rpm (low speed)].
7. Incubate the C-18 stage tips for 5 min at room temperature and centrifuge at maximum 2,000× *g* for 5 min. Reload the flowthrough solution to make sure that as many peptides as possible bind to the C-18 material. For this, pipette again the peptide flowthrough solution onto the C-18 material and repeat the same centrifugation step.
8. Wash C-18 stage tips twice with 100 μL of 0.1% FA and centrifuge for 2 min at maximum speed.
9. Place the tips into a 1.5 mL low binding tube and elute peptides with 70% ACN 0.1% FA.
10. Centrifuge the C-18 stage tips at 2,000× *g* for 5 min to elute the peptides from the C-18 material.
11. Finally, dry peptides in a speed vac and store at -20 ° C before resuspending in 0.1% FA for further LC–MS/MS analysis.

**LC–MS/MS**

Peptide samples are dissolved in 20 μL of 0.1% (v/v) FA. Approximately 1 μg of peptide solution is used for each LC–MS/MS analysis. All LC–MS/MS experiments are performed using a Dionex Ultimate 3000 RSLC nanoUPLC system and a Q Exactive Orbitrap mass spectrometer.

1. Perform separation of peptides by reverse-phase chromatography at a flow rate of 300 nL/min using a reverse-phase nano Easy-spray column (PepMap C18, 2 μm particle size, 100 Å pore size, 75 μm i.d. × 50 cm length). Load peptides onto a pre-column (PepMap 100 C18, 5 μm particle size, 100 Å pore size, 300 μm i.d. × 5 mm length) via the Ultimate 3000 nanoUPLC autosampler with 0.1% FA for 3 min at a flow rate of 15 μL/min.
2. After loading, switch the column valve to allow elution of peptides from the pre-column onto the analytical column. Solvent A is 0.1% FA in water and solvent B is 80% ACN, 20% water, and 0.1% FA. The linear gradient employed is 2%–40% B in 90 min (the total run time including column washing and re-equilibration is 120 min). In between runs, wash the pre-column and analytical column at least four times to avoid carryover.
3. Ionize the LC eluant by means of an Easy-spray source. An electrospray voltage of 2.1 kV is applied in order to ionize the eluant. All m/z values of eluting ions are measured in an Orbitrap mass spectrometer, set at a resolution of 35,000, and scanned between m/z 380 and 1,500.
4. Data-dependent scans (Top 20) are employed to automatically isolate and generate fragment ions by higher energy collisional dissociation [HCD; normalized collision energy (NCE): 25%] in the HCD collision cell, and measurement of the resulting fragment ions are performed in the Orbitrap analyzer, set at a resolution of 17,500. Singly charged ions and ions with unassigned charge states are excluded from being selected for MS/MS and a dynamic exclusion of 20 s is employed.

## Data analysis

Protein identification is performed using Sequest HT or Mascot search engine software operating in Proteome Discoverer 2.3 or above (Eng et al., 1994; Koenig et al., 2008), with the following parameters:

1. Trypsin is set as the enzyme of choice.
2. Precursor ion mass tolerance 20 ppm.
3. Fragment ion mass tolerance 0.1 Da.
4. Maximum of two missed cleavage sites.
5. A minimum peptide length of six amino acids.
6. Fixed cysteine static modification by carbamidomethylation.
7. Variable modification by methionine oxidation & deamidation of asparagine and glutamine.
  Data analysis is performed using open-source Bioconductor packages using R and RStudio (Ihaka and Gentleman, 1996; Rstudio Team, 2020; Gatto et al., 2021). Alternatively, the MaxQuant can be used for the identification of proteins using the Andromeda search engine (Tyanova et al., 2016a). Data can then be assessed and visualized in Perseus (Tyanova et al., 2016b).

**Expected result**

This protocol describes an approach to study the interaction of native membrane proteins with different ligands, like drug molecules. Membrane proteins are known to be difficult to extract in their native conformation, and therefore the development of a method that allows the successful enrichment of native membrane proteins for downstream analysis is of great importance. One of these downstream analyses could be, for example, a protein’s interaction with different ligands and the better characterization of where these molecules bind to the membrane protein. Our protocol describes the enrichment by affinity beads of native nAChRs in SMALP preparations, and as expected, resulting subunits of these receptors can be identified by mass spectrometry. This helps to better characterize receptor–ligand interactions, and our protocol can be applied to various research questions, in a variety of different organisms.

## Recipes

1. **Isotonic lysis buffer**
  0.25 M sucrose, 50 mM Tris-HCl pH 7.4, 10 mM HEPES pH 7.4, 2 mM EDTA, protease inhibitor
2. **SMALP solution**
  5% styrene maleic acid copolymer, 3:1 average ratio of styrene to maleic acid repeat units, 5 mM Tris-Base, 0.15 mM NaCl, pH 8.0
3. **Coupling buffer**
  1. M NaHCO_3_, 0.5 M NaCl, pH 8.3
4. **Tris-buffer or TBS**
  50 mM Tris, 150 mM NaCl, pH 8.0
5. **Tris-buffer or TBS-T**
  50 mM Tris, 150 mM NaCl, pH 8.0 and add to 500 mL, 1,000 μL Tween 20
6. **Blocking solution**
  Marvel dried skimmed milk (5% solution) in TBS-T
7. **Laemmli buffer**
  1 M Tris pH 6.8, 10% SDS, 5% glycerol, 2% bromophenol blue
8. **Sample buffer**
  98% HPLC water, 2% acetonitrile, 0.1% formic acid
